# Effect of Tai Chi Yunshou training on the balance and motor functions of stroke patients: a systematic review and meta-analysis of randomized controlled trials

**DOI:** 10.3389/fneur.2023.1178234

**Published:** 2023-05-12

**Authors:** Liying Zhang, Lijuan Zhang, Xiaoming Yu, Huanxia Zhou, Yuwu Ding, Jiening Wang

**Affiliations:** ^1^The Seventh Clinical School of Medicine, Shanghai University of Traditional Chinese Medicine, Shanghai, China; ^2^Department of the Fourth Day Treatment Room, Fujian Cancer Hospital, Fuzhou, China; ^3^Rehabilitation Medical Center, Seventh People’s Hospital of Shanghai University of Traditional Chinese Medicine, Shanghai, China

**Keywords:** stroke, Tai Chi Yunshou exercises, balance function, motor function, meta-analysis, systematic review

## Abstract

**Background:**

There is insufficient evidence on the effect of Tai Chi Yunshou on improving balance and motor function in stroke survivors. Therefore, this systematic review and meta-analysis aimed to evaluate the effect of Tai Chi Yunshou on improving balance and motor function in stroke patients through a comprehensive literature search.

**Methods:**

English and Chinese databases were searched from inception to February 10, 2023, to collect randomized controlled trials (RCTs) investigating the effects of Tai Chi Yunshou on balance and motor function in stroke survivors. Two reviewers independently selected studies meeting eligibility criteria, extracted required data, and assessed the risk of bias using methods recommended by the Cochrane Reviewers’ Handbook. Primary outcomes were balance function and motor function, while secondary outcomes included walking gait and activities of daily living. Review Manager software (version 5.4.1) was used for data analysis.

**Results:**

Among the 1,400 identified records, 12 eligible randomized controlled trials were finally included, with a total of 966 subjects. The results of the meta-analysis showed that the balance function of the experimental group and the control group was assessed using the Berg Balance Scale (MD = 4.87, *p* < 0.001, I^2^ = 90, 95% CI = 4.46–5.28). The motor function assessment of the experimental group and the control group used the Fugl-Meyer Motor Assessment (SMD = 1.11, *p* < 0.001, I^2^ = 94, 95% CI = 0.94–1.28) and Simple Test of Extremity Function (MD = 10.28, *p* < 0.001, I^2^ = 0, 95% CI = 7.89–12.68). Walking ability was measured using the Time-Up and Go Test (MD = −3.22, *p* < 0.001, I^2^ = 83, 95% CI = −3.71–−2.73). Activities of daily living were measured using the Modified Bathel Index (MD = 4.61, *p* < 0.001, I^2^ = 81, 95% CI = 3.61–5.61).

**Conclusion:**

Initial evidence seems to show that Tai Chi Yunshou training can improve the balance and motor function of stroke survivors and further improve walking ability and daily living ability, and the rehabilitation effect may be better than that of conventional rehabilitation training.

**Systematic Review Registration:**

https://www.crd.york.ac.uk/PROSPERO/display_record.php?RecordID=376969, identifier [CRD42022376969].

## 1. Introduction

Stroke is the second most common cause of death and leading cause of disability worldwide (1, 2). According to the World Health Organization (WHO), approximately 15 million people worldwide suffer from stroke every year, more than 5 million die from stroke, and another 5 million are permanently and severely disabled (3). Among long-term survivors after stroke, 70–80% of stroke patients will experience various types of dysfunction (4). Approximately 23–73% of patients fall within 4 to 6 months after stroke, and balance impairment is the biggest risk factor for falls in stroke survivors (5, 6). Balance function refers to the posture state of the human body, and it is the ability to automatically adjust and maintain posture when exercising or receiving external forces (7).

Regular physical exercise is an effective means to improve balance function and prevent falls in stroke patients, but different exercise methods and exercise intensities have different effects on the postural control ability of stroke patients (8). Because Tai Chi can improve the postural control ability of stroke patients, it has gradually attracted the attention of scholars at home and abroad (9). Tai Chi is a traditional Chinese martial art that emphasizes meditation and controlled breathing to improve the practitioner’s balance, postural control, motor coordination, muscle strength and flexibility (10, 11). There are various forms of Tai Chi, and the movements are relatively complicated. The basic form is “Yunshou” and the movements are simple (both upper limbs draw circles from inside to outside in reverse order), which is the essence of Tai Chi (12). During Tai Chi Yunshou training, the movements follow each other up and down and interact left and right to form a coordinated and unified whole body, and the joints and muscles are coordinated and orderly (13). Bilateral active movement is conducive to the functional reorganization and compensation of nerve cells and ultimately the formation of new neural pathways (14). Tai Chi Yunshou training moves slowly, and footwork training includes progress, stepping back, and looking left and right on the premise of physical stability (15). The center of gravity of the body performs a wide range of motions in the three dimensions of front and rear, left and right, and up and down, which can improve the balance function of the human body (16). When practicing, participants must always pay attention to the internal sensation of the human body (17). The three joints of the patient’s hip, knee, and ankle need to complete the transition from virtual to real to stabilize the foot and body to improve the control ability of the lower limbs of stroke patients and improve the motor function of the lower limbs (18).

At present, studies on the intervention effect of Tai Chi Yunshou on the balance and motor function of stroke patients are all randomized controlled trials or study protocols and lack exhaustive evidence-based medicine support (19). Therefore, it is necessary to perform a systematic review and meta-analysis of the existing evidence.

## 2. Methods and analysis

### 2.1. Study registration

We registered this systematic review and meta-analysis at PROSPERO: https://www.crd.york.ac.uk/PROSPERO/display_record.php?RecordID=376969 (registration ID: CRD42022329925). This systematic review and meta-analysis were reported in light of the Preferred Reporting Items for Systematic Review and Meta-Analysis (PRISMA) 2020 statement (20).

### 2.2. Inclusion criteria

We used the PICOS framework to formulate the inclusion criteria as follows:

(1) Population: Included participants were ischemic or hemorrhagic stroke survivors of any age, sex, and disease stage. These stroke survivors all had balance or motor disorders. Brunnstrom stage > III and Lovett stage > 2. (2) Interventions: We only accepted Tai Chi Yunshou as the focus intervention. The treatment group intervention could be Tai Chi Yunshou and routine rehabilitation training (or not). If rehabilitation therapies were used in the Tai Chi Yunshou group, the rehabilitation treatments were the same as those in the control group. (3) Comparison: The control treatment included routine rehabilitation training (occupational therapy, joint range of motion training, joint mobilization techniques, balance training, walking training, etc.), basic treatment (conventional medical treatment, health care and education), Bobath handshake training, rehabilitation nursing, health education and so on. (4) Outcomes: The primary outcomes were the balance function and motor function. The balance function was measured using the Berg Balance Scale (BBS), while motor function was measured using the Fugl-Meyer Motor Function Scale, the Simple Test for Evaluating Hand Function (STEF), and the Wolf Motor Function Test (WMFT). Secondary outcomes included assessment of activities of daily living, walking ability and gait using the Modified Bathel Index (MBI), Timed Up and Go Test (TUGT) and gait spatiotemporal parameters (included walking speed, step width, stride length, stride time, stride time variability, and double-support time), respectively. (5) Studies: Our systematic review and meta-analysis included randomized controlled trials (RCTs) evaluating the effectiveness of Tai Chi Yunshou on balance and motor function that were published in English or Chinese.

### 2.3. Exclusion criteria

The following types of articles were excluded: (1) conference proceedings, (2) review articles, (3) case reports, (4) retrospective studies, (5) papers from which valid outcome data could not be extracted, (6) studies reporting fewer than 20 subjects, (7) repeated literature, and (8) methodological experimental design, animal experiments, systematic reviews, etc.

### 2.4. Search strategy

The English databases searched included PubMed/Medline, Embase, the Cochrane Library and Web of Science, and the Chinese databases included China National Knowledge Infrastructure (CNKI), China Biomedical Literature Service System (CBM), VIP Database and Wanfang Database. The search time limit was from the date of establishment of the database to February 10, 2023, and the languages were limited to Chinese and English. The intervention method was Tai Chi Yunshou, the disease type was stroke, and the research type was randomized controlled trial.

The search terms for TCY included “tai ji” “tai chi” “tai-ji” “tai-chi” “taiji” “taichi” “Yunshou” “cloud hand”; for stroke they included “cerebrovascular accident” “stroke” “cva” “apoplexy” “brain vascular accident” “cerebral infarction” “brain infarction” “cerebral hemorrhage” “hematencephalon” “encephalorrhagia” “subarachnoid hemorrhage.” The logical operators “AND” and “OR” were used. These search terms included controlled vocabulary terms (e.g., Mesh Subject Headings) and free terms depending on the search strategy for each database. The specific search algorithm for each database is provided in the Supplementary Material.

### 2.5. Study selection

All retrieved documents were imported into Endnote (X9), and duplicate documents were eliminated. Two independent reviewers (LJZ and LYZ) screened studies by title and abstract based on the inclusion criteria. The full texts of all potentially relevant studies were downloaded after cross-checking. Downloaded studies were further evaluated and cross-checked independently by 2 reviewers. In case of disagreement, a third reviewer with rich experience and authority was consulted, and finally, a consensus was reached.

### 2.6. Data extraction

Data on the following aspects were independently extracted by two reviewers (LJZ and LYZ): (1) study information: first author, year of publication, country, sample size, and information related to risk of bias (such as randomization and blinding); (2) participant (study level) characteristics: age, sex, duration, and disease stage; (3) details of the experimental group: TCY regimen (type, frequency, duration, etc.) and/or other combined interventions (type, frequency, duration, etc.); (4) details of the control group: comparison protocol and/or other combined interventions (type, frequency, duration, etc.); and (5) outcome information: primary and secondary outcomes. If there were multiple-arm RCTs, we included only data from the arms with interventions relevant to this study.

### 2.7. Study quality assessment

The methodological quality of all included studies was evaluated according to the method recommended by the Cochrane Systematic Reviewer’s Handbook. Each article was evaluated by at least two reviewers. In case of any dispute, a third independent auditor was consulted to resolve the disagreement.

### 2.8. Data analysis

The data of the included studies were quantitatively analyzed using RevMan v5.4 software. Relative risk (RR) was used to analyze the dichotomous classification results. Continuous results in the same unit were analyzed using the mean difference (MD); otherwise, the standardized mean difference (SMD) was used. Uncertainties are presented as 95% confidence intervals (95% confidence interval, 95% CI). I^2^ was used to assess heterogeneity. When I^2^ ≤ 50% and *p* ≥ 0.1, the heterogeneity was small, and a fixed-effects model was used; when I^2^ > 50% and *p* < 0.1, a random-effects model was used. When I^2^ > 75% and *p* < 0.1, the heterogeneity was large, and sensitivity analysis or subgroup analysis was used. Significance level α = 0.05. A sensitivity analysis was conducted using the one-by-one elimination method, that is, to observe the changes in the combined results after eliminating one study’s data for each indicator. The statistical results did not change, indicating that a single study was not the main reason for the heterogeneity of this study. The publication bias of the studies with more than 7 publications was assessed by the funnel plot, and the distribution on both sides of the funnel was symmetrical, suggesting that there was no possibility of publication bias; otherwise, there might be publication bias. The study intervention characteristics and stroke outcomes were tabulated and compared with each integrated plan group. *p* < 0.01 indicates a significant difference in rehabilitation effect.

## 3. Results

### 3.1. Selection process

**Figure 1:**
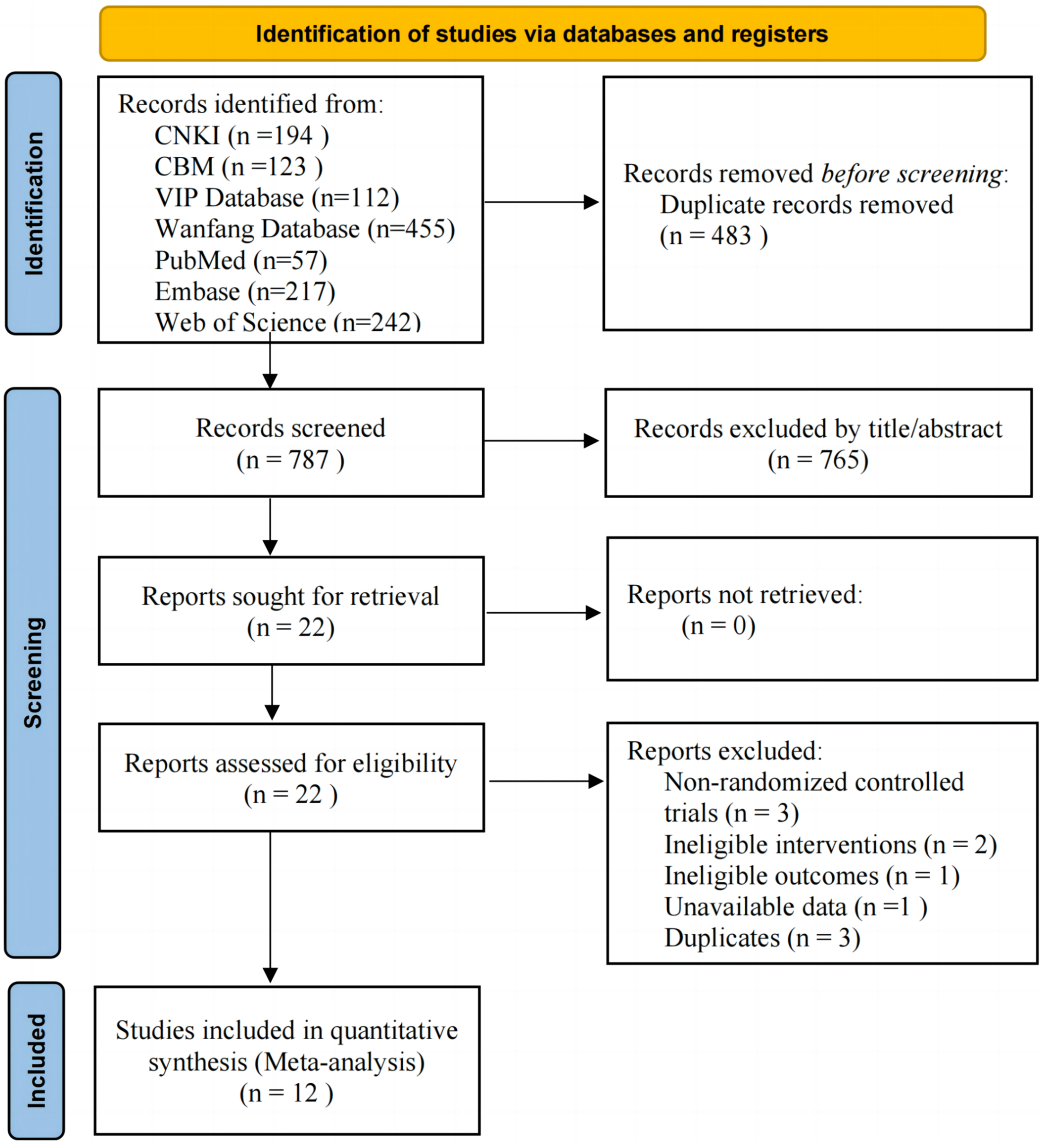
Flow diagram of the study selection process.

We obtained a total of 1,400 records in our literature search. After removing 483 duplicates, we excluded 765 irrelevant records based on title and abstract. The full texts of the 22 remaining records were then assessed, and 12 eligible studies (18, 21–31) were included in the final analysis. The PRISMA flowchart shows the selection procedure (Figure 1).

### 3.2. Basic characteristics of the included studies

All included studies were conducted in China. All included studies showed that participants were under the supervision of 1–2 therapists during Tai Chi Yunshou training to avoid adverse events such as falls. The sample size of the studies ranged from 30 to 244, with a total of 966 participants. The average age of the participants ranged from 47.69 to 73.6 years. Among the 12 studies, 8 of the subjects (21, 23–26, 28, 29, 31) were in the recovery period of stroke (1 month < duration of the disease <6 months), and the subjects of the other 4 studies (18, 22, 27, 30) were in the poststroke sequelae period (duration of the disease >6 months). The experimental arm of 4 studies (18, 23, 27, 30) used Tai Chi Yunshou as monotherapy. The intervention duration was 40 days in 1 study (29), 12 weeks in 4 studies (18, 25, 27, 30) and 8 weeks (2 months) in the remaining studies. Seven studies investigated balance, and eight studies investigated motor function. Table 1 shows the characteristics of the included studies.

**Table 1:** Characteristics of included studies.

References	Sample size (E/C)	Age (E/C)	Gender (F/M)	Disease duration	Intervention			Outcomes
					Methods	Frequence	Duration	
Jiang et al. (21)	60 (30/30)	E: 58.80 ± 11.70 C: 56.46 ± 12.81	E: 7/23 C: 8/22	E: 3.60 ± 1.97 C: 3.30 ± 1.82 (months)	E: TCY C: CRT	5 times/week; 60 min/times	2 months	FMA, STEF
Pang et al. (22)	80 (40/40)	E: 60.49 ± 2.60 C: 60.23 ± 2.81	E: 16/24 C:18/22	E: 1.93 ± 0.25 C: 2.03 ± 0.19 (years)	E: TCY + BPT + CRT C: BPT + CRT	2 times/week; 60 min/times	2 months	BBS
Suzhen (24)	57 (29/28)	E: 58.21 ± 11.44 C: 55.89 ± 13.03	E: 7/22 C: 7/21	E: 3.31 ± 1.85 C: 3.43 ± 1.93 (months)	E: Fixed Step TCY + CRT C: CRT	5 times/week; 30 min/times	8 weeks	FMA, STEF, MBI
Su-zhen (23)	59 (29/30)	E: 59.13 ± 11.45 C: 54.07 ± 12.74	E: 8/21 C: 7/23	E: 3.47 ± 2.40 C: 3.59 ± 2.04 (months)	E:Fixed Step TCY+ Bobath training C: Bobath training	5 times/week; 30 min/times	8 weeks	FMA, STEF, MBI
Xiangbing et al. (18)	30 (14/16)	E: 58.56 ± 8.52 C: 60.71 ± 7.32	E: 5/9 C: 2/14	E: 15.07 ± 8.51 C: 25.31 ± 21.40 (months)	E: TCY C: CRT	5 times/week; 60 min/times	12 weeks	BBS, walking parameters
Xianqiong et al. (25)	132 (66/66)	E: 48.78 ± 13.52 C: 47.69 ± 14.91	E: 24/42 C: 27/39	E: 46.24 ± 27.5 C: 47.66 ± 26.8 (days)	E: TCY + CRT C: CRT	2–7 times/week; 60 min/times	12 weeks	BBS, FMA
Xiaocui et al. (26)	40 (20/20)	E: 56.44 ± 5.82 C: 60.86 ± 6.53	E: 10/10 C: 11/9	E: 49.32 ± 13.80 C: 48.71 ± 13.28 (days)	E: TCY + PST C: PST	5 times/week; 20 min/times	2 months	FMA, MBI
Xie et al. (27)	244 (124/120)	E: 60.9 ± 8.7 C: 60.1 ± 8.6	E: 37/83 C: 25/99	E: 14.5 ± 18.1 C: 14.3 ± 22.1 (months)	E: TCY C: CRT	5 times/week; 60 min/times	12 weeks	BBS, TUGT, FMA, MBI
Xinyu (28)	50 (25/25)	E: 60.92 ± 10.07 C: 60.48 ± 8.29	E: 10/15 C: 9/16	E: 5.50 ± 2.09 C: 5.08 ± 1.56 (months)	E: TCY + CRT C: CRT	5 times/week; 30 min/times	8 weeks	BBS, TUGT, MBI
Xinyuan (29)	60 (30/30)	E: 73.3 ± 8.3 C: 73.6 ± 9.4	E: 8/22 C: 7/23	E: 3.43 ± 1.97 C: 4.43 ± 1.04 (months)	E: TCY + CRT C: CRT	5 times/week; 40 min/times	40 days	WMFT, MBI
Xiuming et al. (30)	60 (30/30)	E: 61.29 ± 1.51 C: 61.35 ± 1.54	E: 8/22 C: 6/24	E: 1.68 ± 0.39 C: 1.71 ± 0.21 (years)	E: TCY C: CRT	2 times/week; 60 min/times	12 weeks	BBS, TUGT
Youbo et al. (31)	94 (47/47)	E: 64.15 ± 9.32 C: 63.23 ± 9.16	E: 17/30 C: 15/32	E: 3.24 ± 1.46 C: 3.11 ± 1.35 (months)	E: TCY + Orem Self-Care C: Orem Self-Care	5 times/week; 60 min/times	8 weeks	BBS, FMA, MBI

E, experimental group; C, control group; F, female; M, male; TCY, Tai Chi Yunshou; CRT, traditional rehabilitation training; BPT, balance pad training; PST, proprioception strengthening training; BBS, Berg balance scale; FMA, Fugl-Meyer assessment; TUGT, time-up-go test; STEF, simple test for evaluating hand function; WMFT, wolf motor function test; MBI, Modifided Bathel index.

### 3.3. Study quality assessment (risk of bias)

**Figure 2:**
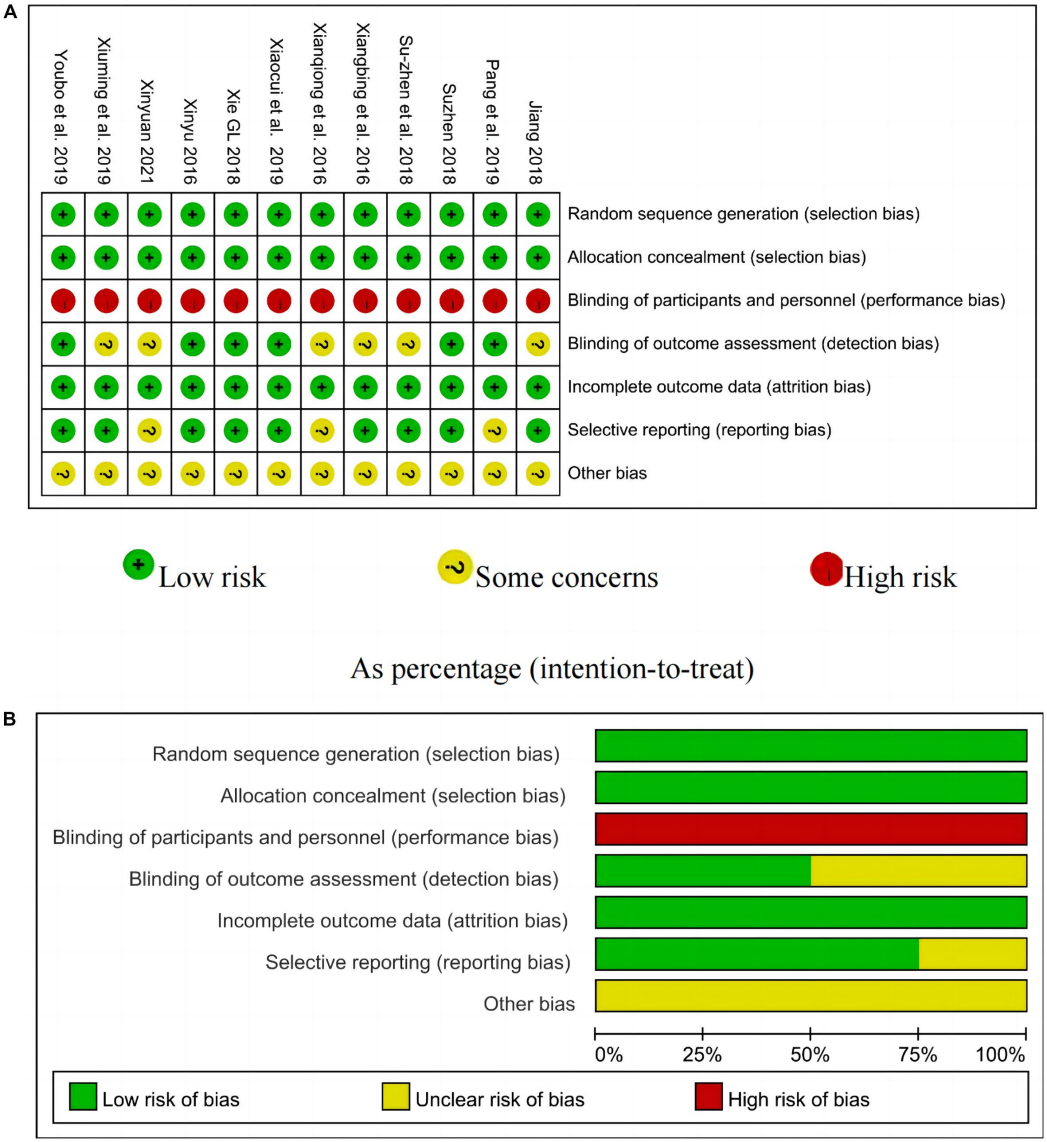
The results of bias assessment. **(A)** Risk of bias individual. **(B)** Overall risk of bias.

All included studies used randomization methods, allocation concealment and attrition bias, but none of them blinded participants. Six studies (21, 22, 24, 26–28) explicitly stated blinding of outcome assessors. All studies reported that baselines were comparable between groups. Five trials (21, 23, 24, 27, 28) provided registration numbers or published protocols, and all trials reported planned results. It was not clear whether there was selective reporting in 3 studies (25, 29, 30). The results for individual study risk of bias and overall risk of bias are shown in Figure 2.

### 3.4. Meta-analysis

#### 3.4.1. Analysis of primary and secondary outcomes

##### 3.4.1.1. Improvement of balance function (assessed using BBS): experimental group vs. control groups

**Figure 3:**
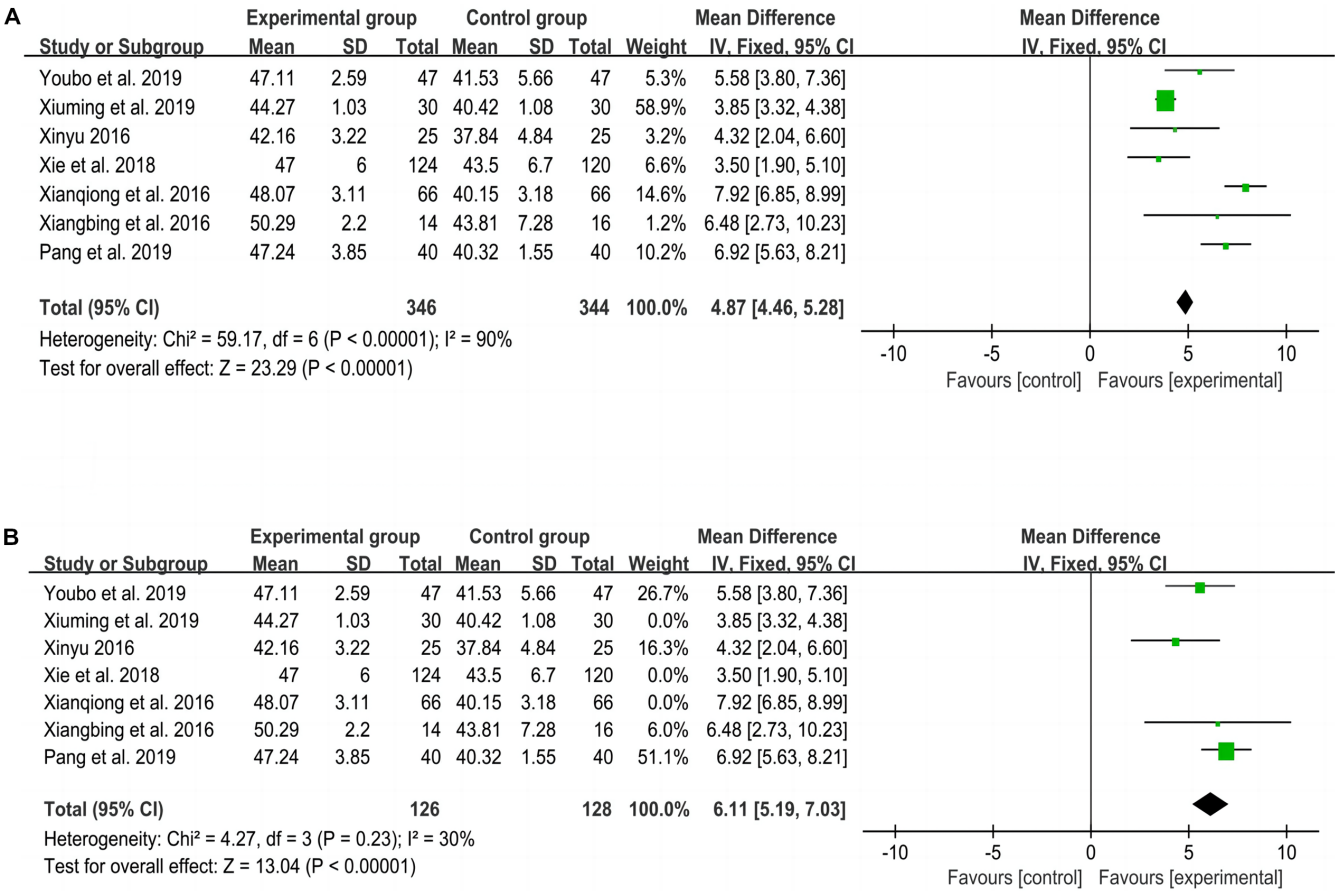
Meta-analysis of balance function between experimental and control groups. **(A)** Meta-analysis of balance function of all included studies. **(B)** Meta-analysis of balance function after excluding 3 studies.

Figure 3A shows the overall meta-analysis of BBS scores between the experimental and control groups. The total number of studies included in the random-effects model was 7, including 690 participants (18, 22, 25, 27, 28, 30, 31). The difference between the experimental group and the control group was significant (MD = 4.87, *p* < 0.001, I^2^ = 90, 95% CI = 4.46–5.28), suggesting that the experimental group had a better effect on improving balance function than the control group.

Due to the high heterogeneity of the BBS results (I^2^ > 75%, *p* < 0.01), to further explore the impact of each article on the total effect size and the accuracy of the results, we performed a sensitivity analysis by excluding one of the studies in turn and then repeated the analysis, as shown in Figure 3B. We found that after excluding 3 studies (25, 27, 30), the heterogeneity decreased (*p* = 0.23, I^2^ = 30%), indicating that these 3 studies were the main source of heterogeneity.

##### 3.4.1.2. Improvement of motor function (assessed using FMA, WMFT, and STEF): experimental group vs. control groups

**Figure 4:**
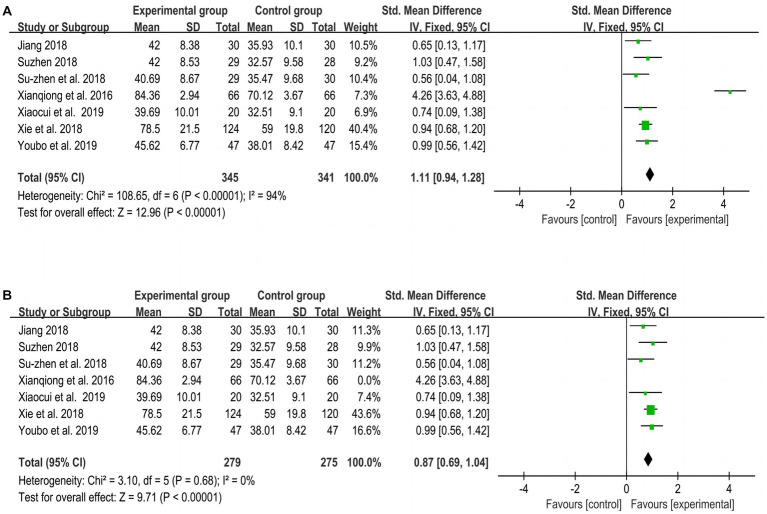
Meta-analysis of FMA between experimental and control groups. **(A)** Meta-analysis of FMA of all included studies. **(B)** Meta-analysis of FMA after excluding 1 study.

**Figure 5:**
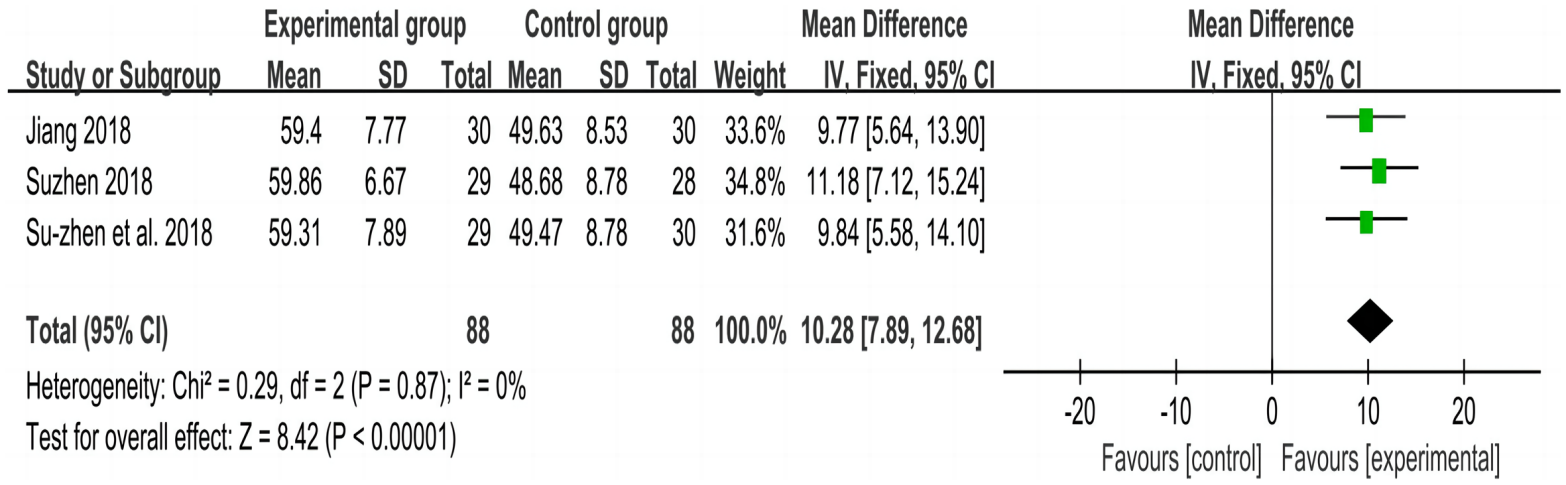
Meta-analysis of STEF of all included studies between experimental and control groups.

Figures 4A, 5 show the overall meta-analysis of FMA and STEF between the experimental and control groups, respectively. Seven studies (21, 23–27, 31) used FMA to assess motor function and found that the improvement effect of the experimental group was better than that of the control group (SMD = 1.11, *p* < 0.001, I^2^ = 94, 95% CI = 0.94–1.28). Three studies (21, 23, 24) using STEF to assess upper limb motor function found that the improvement effect of the experimental group was better than that of the control group (MD = 10.28, *p* < 0.001, I^2^ = 0, 95% CI = 7.89–12.68). An RCT (29) using WMFT to assess upper limb motor function found that the improvement effect of the experimental group was better than that of the control group (t = 1.731, *p* = 0.003).

Due to the high heterogeneity of the FMA results, after excluding one RCT (25), *p* = 0.68 and I^2^ = 0%, indicating that this study was the source of heterogeneity, as shown in Figure 4B.

##### 3.4.1.3. Improvement in ADL (assessed using MBI): experimental group vs. control groups

**Figure 6:**
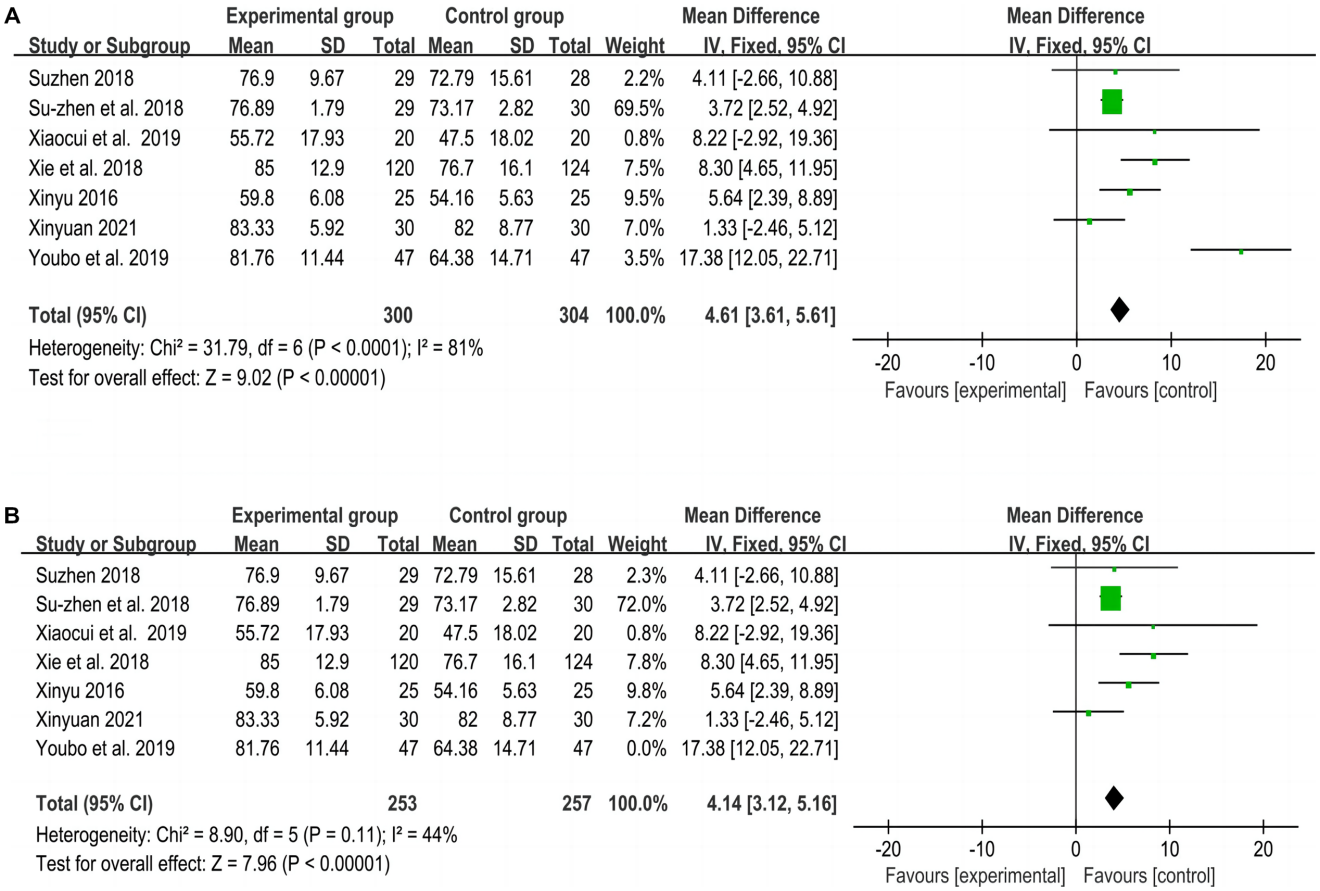
Meta-analysis of MBI between experimental and control groups. **(A)** Meta-analysis of MBI of all included studies. **(B)** Meta-analysis of MBI after excluding 1 study.

Figure 6A shows the overall meta-analysis of MBI between the experimental and control groups, including 7 RCTs (21, 24, 26–29, 31) with a total of 604 subjects. The meta-analysis results found that the improvement effect of the experimental group was better than that of the control group (MD = 4.61, *p* < 0.001, I^2^ = 81, 95% CI = 3.61–5.61).

Due to the high heterogeneity of the MBI results, a sensitivity analysis was performed by excluding one of the studies (31) in turn, and the heterogeneity was found to be smaller (*p* = 0.11, I^2^ = 44%), indicating that this study was the source of heterogeneity, as shown in Figure 6B.

##### 3.4.1.4. Improvement in walking ability and walking gait (assessed using TUGT and walking parameters): experimental group vs. control groups

**Figure 7:**
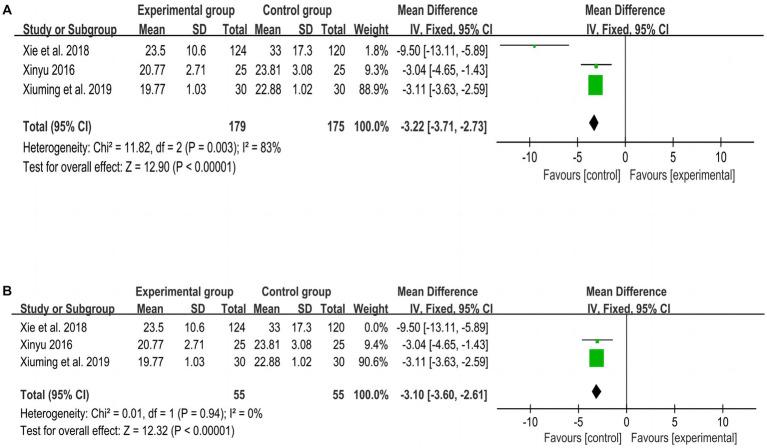
Meta-analysis of TUGT between experimental and control groups. **(A)** Meta-analysis of TUGT of all included studies. **(B)** Meta-analysis of TUGT after excluding 1 study.

Figure 7A shows the overall meta-analysis of TUGT between experimental and control groups, including 3 RCTs (27, 28, 30) with a total of 254 subjects. After the meta-analysis, it was found that the walking ability improvement effect of the experimental group was better than that of the control group (MD = −3.22, *p* < 0.001, I^2^ = 83, 95% CI = −3.71–−2.73). A study using walking parameters to reflect gait showed no significant difference between the experimental group and the control group after treatment (*p* > 0.05).

Because of the high heterogeneity of the TUGT results, a sensitivity analysis was performed by excluding one of the studies (27) in turn, and the heterogeneity was found to be smaller (*p* = 0.94, I^2^ = 0%), indicating that this study was the source of heterogeneity, as shown in Figure 7B.

#### 3.4.2. Publication bias analyses

**Figure 8:**
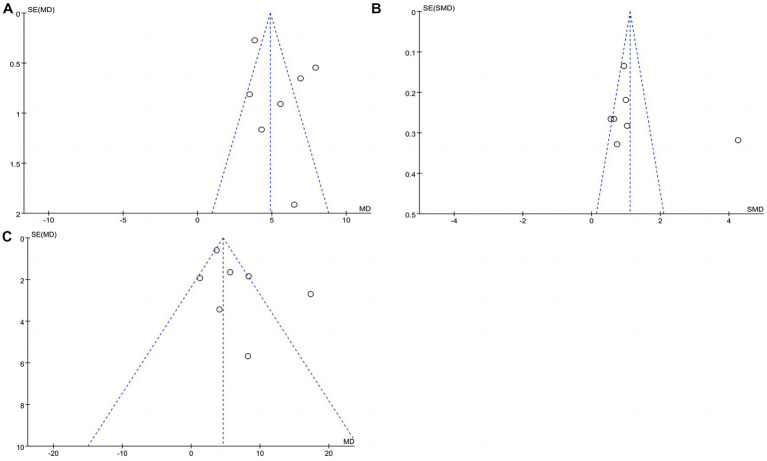
Publication bias between the experimental group and control group. **(A)** Publication bias in BBS; **(B)** Publication bias in FMA; **(C)** Publication bias in MBI.

Figure 8 shows the funnel plots of the 3 evaluation indicators (BBS, FMA and MBI). We performed publication bias analyses on the standardized difference in means between the experimental and control groups. On the BBS, we found that 3 articles (25, 27, 30) fell outside the funnel chart and had large deviations (as shown in Figure 8A). One RCT (25) was found to have a large bias on the FMA (as shown in Figure 8B). One study (31) in the MBI had publication bias, and the other publications were evenly distributed without any evidence of publication bias (as shown in Figure 8C).

### 3.5. Adverse events

None of the 12 articles included in this study reported any adverse events caused by Tai Chi Yunshou training and routine rehabilitation training.

## 4. Discussion

This study systematically reviewed and quantified the differences in balance function and motor function in stroke patients treated with Tai Chi Yunshou (or combined with conventional rehabilitation training) and conventional rehabilitation training. Our meta-analysis showed that stroke patients who received Tai Chi Yunshou training had higher balance function and motor function than those who received conventional rehabilitation training. This difference in balance function and motor function also varies with different evaluation indicators.

### 4.1. The effect of Tai Chi Yunshou on balance function

In this study, through a meta-analysis of the BBS of 7 RCTs, it was found that Tai Chi Yunshou can improve the balance function of stroke survivors, and the effect is better than that of conventional rehabilitation training. Stroke survivors often have impaired limb control and ability to maintain balance, making them less stable than people with similar asymmetrical postures (32, 33). Initial balance dysfunction predicts functional recovery after stroke; thus, balance training is an important aspect of stroke rehabilitation (34). Tai Chi Yunshou is the basic form of various forms of Tai Chi (12). It enables the lower limbs to complete eccentric, centripetal, and isometric contractions in a specific posture through the half-squat posture and the role of single-leg support and continuously switches between these types of muscle contractions, increasing the strength of the lower limbs (35). The muscle strength of the hip joint, knee joint, and ankle joint also promotes the body’s own ability to control the lower limbs (36). In the process of Tai Chi Yunshou training, the transfer of the center of gravity to and from the lower limbs is combined with the left and right arc movements of the upper limbs, and the eyes go hand in hand, which not only improves the support and control ability of the limbs but also exercises the visual spatial adaptation ability of stroke patients, thereby further improving their balance function (37). The center of gravity of the body performs a wide range of motions in the three dimensions of front and rear, left and right, and up and down, which can promote the improvement of the body’s balance function (38).

### 4.2. The effect of Tai Chi Yunshou on motor function

Eight RCTs in the literature included in this study showed that Tai Chi Yunshou can not only improve the motor function of stroke survivors, especially the motor function of the upper limbs, but also outperform conventional rehabilitation training. On the frontal plane, upper body movements help maintain balance during walking (39). Stroke survivors are therefore encouraged to perform arm exercises for balance (40). Through the cooperation of both upper limbs, with the trunk as the axis, the upper limb of the affected side draws a circle in a clockwise upward motion, and the healthy side draws a circle in a counterclockwise upward motion; at the same time, it trains shoulder joint abduction, external rotation, forward flexion, elbow flexion, pronation and supination of the forearm and circular motion of the wrist joint (17, 41). Compared with single-joint training, Tai Chi Yunshou requires the body to move coherently, which can enhance proprioception, stimulate the motor sensory area of the brain, and provide good motion feedback (42, 43). The active movement of the upper and lower limbs and the interaction between the left and right can improve the blood collateral circulation of the brain, reshape and compensate for the nerve function of the brain, and then promote the recovery of motor function (11, 44). Tai Chi Yunshou is also a kind of isotonic exercise that can exercise the joints and ligaments of the whole body and strengthen muscle strength (45).

### 4.3. The effect of Tai Chi Yunshou on walking ability and gait

The ability to maintain balance has a strong influence on walking ability, and trunk balance in particular is a determinant of motor function in stroke patients (46). In this study, 3 RCTs of TUGT were used to evaluate walking ability, and Tai Chi Yunshou had a significant effect and was superior to conventional rehabilitation training. The movement of the joints of the lower limbs is a closed-chain movement in the Tai Chi Yunshou, which effectively stimulates the joint proprioceptors, improves the response-ability, and can improve the static balance in the standing position and the dynamic balance during walking, whether it is nerve conduction velocity, movement strategy generation, or protective action (47, 48). Improvement in the generation of walking thereby reduces standing and walking time and enhances functional walking ability (28).

The footwork training of Tai Chi Yunshou includes progress, regress, and looking left and right under the premise of physical stability (40). Studies by Xiangbin et al. (18) have shown that Tai Chi Yunshou can improve the walking gait of stroke survivors in the standing phase and supporting phase, and the effect is similar to that of conventional walking rehabilitation training.

### 4.4. The effect of Tai Chi Yunshou on activities of daily life

The 7 studies included in this study show that Tai Chi Yunshou can improve daily life, and the effect is better than that of conventional rehabilitation training. The goal of clinical rehabilitation for stroke patients is to maximize the function of patients such that patients can take care of themselves, achieve self-care, and return to family and society (24). Studies (24, 49) have shown that even without intervention, the motor function of stroke patients changes over time, and the self-care ability of patients also improves with the improvement in their motor function. Whether balance function is improved or motor function is improved, the patient’s self-care ability will also be improved (50–52). In the process of Tai Chi Yunshou training, consciousness, breathing and movement are coordinated and unified, which can effectively relieve tension and depression (53). The movements of Tai Chi Yunshou are similar to those of drinking water, wiping your face, combing your hair, etc. It is beneficial to exercise daily life skills in the actual environment (24).

### 4.5. Limitations

Our study has some limitations. First, although as many documents as possible were retrieved, it is still possible that some studies missed detection. Second, at present, there are few randomized controlled studies on the intervention of Tai Chi Yunshou training in stroke at home and abroad, and the quality of the literature is average, which may limit the judgment of the results. Third, we only included articles published in Chinese or English, which may have resulted in language bias. Fourth, no studies have been conducted on the effects of Tai Chi Yunshou training on stroke patients at different stages. Fifth, there may be a risk of falls in Tai Chi Yunshou training, and this study did not evaluate the risk of falls. In the future, we need large-sample, multicenter, high-quality randomized controlled trials and more systematic and comprehensive indicators to evaluate the efficacy of Tai Chi Yunshou on the dysfunction of poststroke patients. As water is a unique medium that allows mobility and stability exercises with a decreased risk of falling (54, 55), Tai Chi Yunshou training might be tested in water. Sixth, the control treatments applied in the studies analysed here were heterogenous, so the superiority of Tai Chi Yunshou needs be confirmed in prospective controlled studies.

## 5. Conclusion

Although only 12 studies were included, the initial evidence seems to show that Tai Chi Yunshou training can improve the balance and motor functions of stroke survivors, further improve walking ability and daily living ability, and the rehabilitation effect may be better than that of conventional rehabilitation training. Therefore, Tai Chi Yunshou training should be further developed for stroke patients on the basis of further clinical studies. Research in Tai Chi Yunshou for additional neurological disorders is also required to establish appropriate preventive–rehabilitative programs.

## Author contributions

LiyZ, LijZ, and JW set the theme. LijZ, YD, and HZ retrieved and evaluated these studies. LiyZ and LijZ extracted and analyzed the data. LiyZ and XY wrote the manuscript. JW, HZ, and XY supervised the whole process and made the final decision. All authors contributed to the article and approved the submitted version.

## Funding

This study was funded by the Shanghai Health Commission Accelerating the Development of Traditional Chinese Medicine Three-Year Action Plan Project [Grant No. ZY (2021–2023)-0104-01], Shanghai Municipal Health and Health Commission Chinese Medicine Research Project (Grant No. 2020LP004), and Pudong New Area “National Traditional Chinese Medicine Development Comprehensive Reform Pilot Zone” construction project (Grant No. PDZY-2022-0702).

## Conflict of interest

The authors declare that the research was conducted in the absence of any commercial or financial relationships that could be construed as a potential conflict of interest.

## Publisher’s note

All claims expressed in this article are solely those of the authors and do not necessarily represent those of their affiliated organizations, or those of the publisher, the editors and the reviewers. Any product that may be evaluated in this article, or claim that may be made by its manufacturer, is not guaranteed or endorsed by the publisher.
