# Supplementary material for: Effect of Tai Chi Yunshou training on the balance and motor functions of stroke patients: a systematic review and meta-analysis of randomized controlled trials
Source: Front Neurol. 2023 May 12;14:1178234. doi: 10.3389/fneur.2023.1178234 (PMC10213663; doi:10.3389/fneur.2023.1178234)
Supplement: Supplementary file 1 [file Data_Sheet_1.doc]

**Search strategies of all databases**

| **Database** | **Search strategies** |
| --- | --- |
| PubMed | ("Stroke"[Mesh] OR (cerebrovascular accident[Title/Abstract]) OR (apoplexy[Title/Abstract])) OR (cva[Title/Abstract])) OR (brain vascular accident[Title/Abstract]) OR (cerebral infarction[Title/Abstract]) OR (brain infarction[Title/Abstract])) OR (cerebral hemorrhage[Title/Abstract]) OR (hematencephalon[Title/Abstract]) OR (encephalorrhagia[Title/Abstract]) OR (subarachnoid hemorrhage[Title/Abstract]) AND ("Tai Ji"[Mesh]) OR (Tai Chi[Title/Abstract]) OR (Tai-chi[Title/Abstract]) OR (Tai-ji[Title/Abstract]) OR (taiji[Title/Abstract]) OR (taichi[Title/Abstract]) OR (Yunshou[Title/Abstract]) OR (cloud hand[Title/Abstract]) |
| EMbase | #1 'Stroke'/exp OR 'cerebrovascular accident':ab,ti OR 'apoplexy':ab,ti OR 'cva':ab,ti OR 'brain vascular accident':ab,ti OR 'cerebral infarction':ab,ti OR 'brain infarction':ab,ti OR 'cerebral hemorrhage':ab,ti OR 'hematencephalon':ab,ti OR 'encephalorrhagia':ab,ti OR 'subarachnoid hemorrhage':ab,ti  #2 'Tai Ji'/exp OR 'Tai Chi'/exp OR 'Tai-chi':ab,ti OR 'Tai-ji':ab,ti OR 'taiji':ab,ti OR 'taichi':ab,ti OR 'Yunshou':ab,ti OR 'cloud hand':ab,ti OR 'Tai Chi Chun':ab,ti  #3 #1 AND #2 |
| The Cochrane library | #1 MeSH descriptor: [stroke] explode all trees  #2 (cerebrovascular accident):ti,ab,kw OR (apoplexy):ti,ab,kw OR (cva):ti,ab,kw OR (brain vascular accident):ti,ab,kw OR (cerebral infarction):ti,ab,kw OR (brain infarction):ti,ab,kw OR (cerebral hemorrhage):ti,ab,kw OR (hematencephalon):ti,ab,kw OR (encephalorrhagia):ti,ab,kw OR (subarachnoid hemorrhage):ti,ab,kw  #3 #1 OR #2  #4 MeSH descriptor: [Tai Ji] explode all trees  #5 (Tai Chi):ti,ab,kw OR (Taichi):ti,ab,kw OR (Taiji):ti,ab,kw OR (Yunshou):ti,ab,kw OR (cloud hand):ti,ab,kw OR (Tai-ji):ti,ab,kw OR (Tai-chi):ti,ab,kw  #6 #4 OR #5  #7 #3 AND #6 |
| Web of Science | #1 ((TS=(stroke) OR TS=(cerebrovascular accident) OR TS=(cva) OR TS=(apoplexy) OR TS=(brain vascular accident) OR TS=(cerebral infarction) OR TS=(brain infarction) OR TS=(cerebral hemorrhage) OR TS=(hematencephalon) OR TS=(encephalorrhagia))  #2 ((TS=(Tai Ji Yunshou ) OR TS=(Tai Ji) OR TS=(Tai Chi) OR TS=(Taiji) OR TS=(Taichi) OR TS=(Tai-ji) OR TS=(Tai-chi) OR TS=(Yunshou)) OR TS=(cloud hand))  #3 #1 AND #2 |
| China National Knowledge Infrastructure (CNKI) | ((主题=脑卒中) OR (主题=中风) OR (主题=脑出血) OR (主题=脑溢血) OR (主题=脑血栓) OR (主题=脑梗死) OR (主题=脑血管病) OR (主题=脑血管意外)) AND ((主题=太极) OR (主题=太极拳) OR (主题=太极云手) OR (主题=云手) OR (主题=太极拳云手))  [( ( ( ( ( ( ( ( ( 主题%='脑卒中' or 题名%='脑卒中' ) OR ( 主题%='中风' or 题名%='中风' ) ) OR ( 主题%='脑出血' or ...](https://kns.cnki.net/kns8/Manage/Search.html?id=525) |
| Wanfang Database | #1 主题:(卒中) or 主题:(脑卒中) or 主题:(中风) or 主题:(脑出血) or 主题:(脑溢血) or 主题:(脑梗死) or 主题:(穴脑血栓 or 主题:(脑血管病) or 主题:(脑血管意外)  #2 主题:(太极) or 主题:(太极拳) or 主题:(太极云手) or 主题:(云手) or 主题:(太极拳云手)  #3 #1 AND #2 |
| Chinese Science and Technology Periodical Database (VIP) | (M=卒中 OR M=脑卒中 OR M=中风 OR M= 脑出血 OR M=脑梗死 OR M= 脑溢血 OR M= 脑血管病 OR M=脑血管意外) AND (M=太极 OR M=太极拳 OR M=太极云手 OR M=云手 OR M=太极拳云手) |
| Chinese Biomedical Literature Database (CBM) | #1 "卒中"[不加权:扩展]  #2 "脑卒中"[常用字段:智能] OR "中风"[常用字段:智能] OR "脑梗死"[常用字段:智能] OR "脑出血"[常用字段:智能] OR "脑溢血"[常用字段:智能] OR "脑血管病"[常用字段:智能] OR "脑血管意外"[常用字段:智能]  #3 "太极"[不加权:扩展]  #4 "太极拳"[常用字段:智能] OR "太极云手"[常用字段:智能] OR "太极拳云手"[常用字段:智能] OR "云手"[常用字段:智能]  #5 #2 OR #1  #6 #4 OR #3  #7 #5 AND #6 |
